# Ethnomedicines used in Trinidad and Tobago for urinary problems and diabetes mellitus

**DOI:** 10.1186/1746-4269-2-45

**Published:** 2006-10-13

**Authors:** Cheryl A Lans

**Affiliations:** 1 BCICS, University of Victoria, British Columbia, V8W 2Y2, Canada

## Abstract

**Background:**

This paper is based on ethnobotanical interviews conducted from 1996–2000 in Trinidad and Tobago with thirty male and female respondents.

**Methods:**

A non-experimental validation was conducted on the plants used for urinary problems and diabetes mellitus: This is a preliminary step to establish that the plants used are safe or effective, to help direct clinical trials, and to inform Caribbean physicians of the plants' known properties to avoid counter-prescribing.

**Results:**

The following plants are used to treat diabetes: *Antigonon leptopus*, *Bidens alba*, *Bidens pilosa*, *Bixa orellana*, *Bontia daphnoides*, *Carica papaya*, *Catharanthus roseus*, *Cocos nucifera*, *Gomphrena globosa*, *Laportea aestuans*, *Momordica charantia*, *Morus alba*, *Phyllanthus urinaria *and *Spiranthes acaulis*. *Apium graviolens *is used as a heart tonic and for low blood pressure. *Bixa orellana*, *Bontia daphnoides*, *Cuscuta americana *and *Gomphrena globosa *are used for jaundice. The following plants are used for hypertension: *Aloe vera*, *Annona muricata*, *Artocarpus altilis*, *Bixa orellana*, *Bidens alba*, *Bidens pilosa*, *Bonta daphnoides*, *Carica papaya*, *Cecropia peltata*, *Citrus paradisi*, *Cola nitida*, *Crescentia cujete*, *Gomphrena globosa*, *Hibiscus sabdariffa*, *Kalanchoe pinnata*, *Morus alba*, *Nopalea cochinellifera*, *Ocimum campechianum*, *Passiflora quadrangularis*, *Persea americana *and *Tamarindus indicus*.

The plants used for kidney problems are *Theobroma cacao*, *Chamaesyce hirta*, *Flemingia strobilifera*, *Peperomia rotundifolia*, *Petiveria alliacea*, *Nopalea cochinellifera*, *Apium graveolens*, *Cynodon dactylon, Eleusine indica, Gomphrena globosa*, *Pityrogramma calomelanos *and *Vetiveria zizanioides*. Plants are also used for gall stones and for cooling.

**Conclusion:**

*Chamaesyce hirta*, *Cissus verticillata*, *Kalanchoe pinnata*, *Peperomia *spp., *Portulaca oleraceae*, *Scoparia dulcis*, and *Zea mays *have sufficient evidence to support their traditional use for urinary problems, "cooling" and high cholesterol.

Eggplant extract as a hypocholesterolemic agent has some support but needs more study. The plants used for hypertension, jaundice and diabetes that may be safe and justify more formal evaluation are *Annona squamosa*, *Aloe vera*, *Apium graveolens*, *Bidens alba*, *Carica papaya*, *Catharanthus roseus*, *Cecropia peltata*, *Citrus paradisi*, *Hibsicus sabdariffa*, *Momordica charantia*, *Morus alba*, *Persea americana*, *Phyllanthus urinaria*, *Tamarindus indicus *and *Tournefortia hirsutissima*. Several of the plants are used for more than one condition and further trials should take this into account.

## Background

Latin American and Caribbean policy makers have recognised that many problems in primary health care are due to lack of knowledge and sensitivity to local health practices, and to the economic and cultural factors associated with these practices [1]. However available herbal products have no clear statement of content; or medically related information on the package labels, and have not been validated or certified by any recognised body. This concerns consumers (potential and actual) and medical practitioners who may unknowingly counter-prescribe these herbal products [1]. Not all of the plants reported to be useful are harmless. These considerations (among others) underlie the study of ethnomedicinal plants used in Trinidad and Tobago from 1995 to 2000 [1]. This paper focuses on the plants used for diabetes, urinary problems and related problems. The World Health Organization has projected that the incidence of type 2 diabetes in Trinidad and Tobago will increase from 70,000 in the year 2000 to 89,000 by the year 2010 [2]. Type 1 diabetes typically affects less than 10% of patients [3]. Both types of diabetes involve hyperglycemia and several grave complications resulting from long-term hyperglycemia [3]. Control of the glucose levels in the blood is the most effective treatment. Long term reductions in hyperglycemia reduce the likelihood of developing microvascular and macrovascular complications [3]. Type 2 diabetes mellitus has both genetic and environmental components [2]. Previous studies (including Caribbean studies) of offspring or first-degree relatives of patients with type 2 diabetes have confirmed that insulin resistance and/or hyperinsulinaemia is the antecedent biochemical marker for developing diabetes in middle age [2]. First-degree relatives of diabetic patients are more likely to develop diabetes and also have an increased cluster of biochemical metabolic syndrome risk factors [2]. Although insulin resistance is independently associated with obesity, it is more severe in obese patients (80% of type 2 diabetic patients in the West) [3]. Diabetic patients typically have delayed or impaired wound healing, and may develop chronic ulcers [4]. Diabetic ulcers of the lower limbs and feet are associated with high morbidity and amputation is a common treatment. Peripheral neuropathy and peripheral vascular disease are possible underlying factors in diabetic wound formation, but dermal atrophy is a possible contributing factor [4]. Reduced fibroblast growth, increased expression of matrix-degrading matrix metalloproteinases (MMPs), and decreased matrix synthesis are consequences of chronic vascular disease in diabetic skin as well as in other situations characterised by peripheral vascular disease [4]. Atrophic skin is more likely to develop wounds than healthy skin. In addition, insufficient extracellular matrix production during the proliferative phase of wound repair contributes to poor healing [4].

A 1990 Caribbean study on folk medicine used for diabetes mellitus found no difference between non-users of informal medication and those who used it in addition to, or in replacement of, formal medication to control diabetes mellitus in Jamaica [5]. The authors found that teas made from periwinkle (*Catharantus *species) and rice bitters (*Andrographics paniculata*) interfered with the control of diabetes mellitus. In a previous study by one of the authors, with more severe cases of diabetes, formal medication gave better control of the diabetes than folk medicine teas [6].

A more recent study conducted in Trinidad and Tobago examined 622 people with diabetes mellitus. Herbal remedies for diabetes were used by 152 (24%) of patients [7]. Caraaili (*Momordica charantia*), aloes (*Aloe vera*), olive bush (*Bontia daphnoides*) and seed-under-leaf (*Phyllanthus urinaria*) were the plants most frequently used. Patients who reported burning or numbness in the feet or feelings of tiredness, weakness, giddiness or dizziness used folk medicines more frequently than those who reported other symptoms [7]. Subsequent studies by the same authors have discarded folk medicine and focussed on the standards of conventional medical care for diabetes [8-10]. In a 2004 study they found that diabetes was associated with low income status and worse health status and more frequent expenditure on medical services [9]. Although the authors of this study found that the control group had access to health insurance this access is not common in Trinidad. In an earlier paper the same authors estimated the annual cost of hospital admissions with diabetes at TT10.66 million (UK 1.24 million pounds) [10], type 2 diabetes is a costly and largely avoidable problem.

In the late 1980s an IDRC-funded study examined the prevalence of diabetes and hypertension in women in Trinidad [11]. They screened 4013 women older than 25 years in 3152 households and 769 had been diagnosed with diabetes mellitus or hypertension. They concluded that the women's conditions were not managed adequately by the health system and that they should assume greater responsibility for managing their own health [11]. It is therefore puzzling that little research has been conducted on the medicinal plants used for diabetes and their efficacy.

Instead studies have examined ethnic or gender differences in prevalence of diabetes mellitus or compliance with conventional treatment protocols [12]. Finding alternatives is important because 10 – 20% of the population is affected. The leading causes of death and illness for the past thirty years in Trinidad and Tobago have been cardiovascular disease, cerebrovascular disease, cancer, hypertension and diabetes mellitus [13]. In a 2002 study, 2117 randomly selected patients from 35 randomly selected government health centres in Trinidad were found to be suffering from diabetes-related foot problems. Of these, 49% had burning or numbness in the feet, 1241 (59%) self-reported visual impairment and 12% had foot ulceration of whom 92 (4%) had major or minor amputation [14].

This author conducted ethnoveterinary research in Trinidad and Tobago from 1995 to 2000. During this research many ethnomedicinal plant uses were discovered that did not have comparative uses for animals. This paper deals with the twenty-two plants used for hypertension, the four plants used for jaundice and the fourteen used for diabetes. It also presents the forty-five plants for urinary problems, "cooling" and high cholesterol levels.

A substantial body of research published outside the Caribbean since 2000 has provided sufficient data to conduct a preliminary evaluation of the researched plants and this evaluation is presented in the discussion section of this paper [15]. Several (approx. 30) of the plant uses have already been recorded in an ethnomedicinal study in one rural community in the northern range of Trinidad [16] and in the study on diabetes mellitus mentioned previously [7]. Several older studies referred to in the discussion section of the paper were reviewed previously and their original references will not be given for space reasons [1].

## The Trinidadian hot/cold system

Caribbean folk medicine is a marriage-α-cinq:European folkmedicine, scientific medicine, African-based practices, Amerindianmedicine and Indian-based medicine, a product of inter-group borrowing or medical syncretism [1]. Activities, food and medicines are classified in various ethnomedicinal systems as hot or cold. The Trinidadian hot/cold system is not humoral in the sense that balance must be established between hot and cold, it is cathartic in that remedies are taken to remove heat from the system [1]. Some researchers consider that the hot-cold concept of health and illness is absent in Spanish folk medicine and did not exist at the folk level in the past [1]. These beliefs are now widespread in Latin America and the Caribbean, and according to G.M. Foster were derived from the élite and scholarly Hippocratic-galenic traditions that were brought to the Spanish colonies by Spanish physicians and clergy [17,18]. The hot-cold valence refers to the traditional belief that heat opens the body and facilitates the blood's free flow, whereas cold causes the blood to stop flowing and clog the arteries, veins and womb [1,17,18]. Heat comes from the sun, work, sleeping, burns, cooking, and reproductive activities. Linked to the hot/cold dichotomy is a system of blood beliefs where an excess or lack of cold or heat in the body through exposure or diet causes illness. Blood then becomes 'bad' or dirty [1]. Female *Warao *herbalists in Eastern Venezuela also use the concept of bad blood [19].

Teas are used for 'cooling' if there is too much 'heat' in the body. Cooling teas are used prophylatically when they are taken to keep the body healthy by cooling the 'system', or the bladder, meaning that they remove the 'heat' or impurities in the system [1,17-20]. Cooling teas become treatment when they are taken for undiagnosed or unspecified illnesses or when feeling unwell. Purges reduce the heat further and 'clean the blood'. Thereare also 'hot' plants to stimulate the blood or to treat 'cold' illnesses, and 'hot' external applications like 'soft candle' 'grated nutmeg' and hot poultices [1,17-20]. Medicines are administered in accordance with the identity between cause and effect. Expulsion of disease-causing impurities is the primary mechanism by which bodily equilibrium is restored. Folk medicines achieve cures through 'bitterness', 'cutting', 'cooling', 'building', 'purging or washing out', and 'drawing out' [1,17-20].

## Methods

This study adhered to the research guidelines and ethical protocols of Wageningen University in the Netherlands. Thirty respondents, ten of whom were male were interviewed from September 1996 to September 2000. The respondents were obtained by snowball sampling, and were found in thirteen different sites, 12 in Trinidad and one in Tobago. Snowball sampling was used because there was no other means of identifying respondents and because the research covered the entire island-area rather than concentrating on one village. The chief objective of the sampling method was to identify knowledgeable respondents; no priority was given to extrapolating the data to the wider population to establish prevalence of use. No statistical analysis was applied to the data.

Twenty respondents were interviewed once, the other ten (who were healers) were interviewed three or four times. Healers were also asked to reconstruct the circumstances and contexts of the plant uses so that the means of administration of the plants could be identified. No interview schedule of questions was used but a more qualitative, conversational technique. Plants were collected when available to verify that the common names used by each respondent were the same in each ethnic group as those recorded in the literature. The majority of the plants were identified at the Herbarium of the University of the West Indies but voucher samples were not deposited. This ethnomedicinal study was part of a larger research project on ethnoveterinary medicine; other data collecting techniques were used in the larger study [1].

## Validation of practices

A preliminary validation of ethnomedicinal practices is considered a preliminary step to establish which plants are safe or effective and which uses should be discontinued. It also ensures that clinical trials are not wasted on plants that are used for cultural or religious reasons. This is important not only because of the waste of time, money and energy, but because negative results can lead to the discrediting of further effort [1].

Anthropologists like Posey [21] and Hastrup and Elsass [22] claim that anthropologists should not decide whether indigenous beliefs and practices are or are not scientific as this has colonial overtones. Other anthropologists claim that indigenous knowledge systems represent the cultural dimension of development and cannot be reduced to the empirical knowledge that they contain [23]. These anthropological reservations have some value; however validation of traditional medicines is important since most scientists will not use local medicines without some form of validation and they have discouraged the use of these medicines leading to their disuse and eventual loss [1]. A framework of indigenous/ethnoveterinary knowledge that can interface with science and technology is more likely to influence scientific research agendas and development work [1,24].

The validation of the remedies was conducted with a non-experimental method [25]. This method consists of:

1. obtaining an accurate botanical identification,

2. determining whether the folk data can be understood in terms of bioscientific concepts and methods,

3. searching the chemical/pharmaceutical/pharmacological literature for the plant's known chemical constituents and to determine the known physiological effects of either the crude plant, related species, or isolated chemical compounds that the plant is known to contain.

This information is used to assess whether the plant use is based on empirically verifiable principles or whether symbolic aspects of healing are of greater relevance. If ethnobotanical data, phytochemical and pharmacological information supports the folk use of a plant species it can be grouped into the validation level with the highest degree of confidence.

Four levels of validity were established [25]:

1. If no information supports the use it indicates that the plant may be inactive; or no research has been done on the plant.

2. A plant (or closely related species of the same genus), which is used in geographically or temporally distinct areas in the treatment of similar illnesses, attains the lowest level of validity, if no further phytochemical or pharmacological information validates the popular use. Use in other areas increases the likelihood that the plant is active against the illness.

3. If in addition to the ethnobotanical data, phytochemical or pharmacological information also validates the use in Trinidad and Tobago, the plant may exert a physiological action on the patient and is more likely to be effective than those at the lowest level of validity.

4. If ethnobotanical, phytochemical and pharmacological data supports the folk use of the plant, it is grouped in the highest level of validity and is most likely an effective remedy.

## Results

Plants used for urinary problems

Forty-five plants were used for urinary problems, "cooling" and high cholesterol levels. The term "stoppage of water" means urinary retention. Four plants were used for bladder problems:*Costus scaber*, *Pilea microphylla*, *Kalanchoe pinnata *and *Cocos nucifera*. Two plants were used for high cholesterol levels: *Solanum melongena *and *Portulaca oleraceae*.*Bauhinia cumanensis/Bauhinia excisa *and *Capraria biflora *were used for gall stones.

Twenty-four plants were used for "cooling": *Musa *species, *Begonia humilis*, *Bontia daphnoides*, *Cissus verticillata*, *Coleus aromaticus*, *Commelina elegans*, *Cuscuta americana*, *Cyperus rotundus*, *Desmodium canum*, *Entada polystachya*, *Justicia pectoralis*, *Momordica charantia*, *Peperomia pellucida*, *Ruellia tuberosa*, *Sansevieria guineensis*, *Stachytarpheta jamaicensis*, *Scoparia dulcis*, *Cassia alata*, *Capraria biflora*, *Kalachoe pinnata*, *Mimosa pudica*, *Lepianthes peltata*, *Tournefortia hirsutissima *and *Solanum americanum*.*Bauhinia cumanensis*, *Bauhinia excisa *and *Capraria biflora *were used for gall stones, while *Hibiscus sabdariffa *was used to "clean the liver and blood".

The following fourteen plants were used for kidney and other urinary problems: *Kalachoe pinnata*, *Mimosa pudica*, *Chamaesyce hirta*, *Flemingia strobilifera*, *Peperomia rotundifolia*, *Petiveria alliacea*, *Nopalea cochinellifera*, *Apium graveolens*, *Cynodon dactylon*, *Zea mays*, *Theobroma cacao*, *Lepianthes peltata*, *Eleusine indica*, *Gomphrena globosa*, *Pityrogramma calomelanos *and *Vetiveria zizanioides*.

The results are summarized in Table 1.

**Table 1:** Ethnomedicinal plants used for "cooling", high cholesterol and urinary problems in Trinidad and Tobago

Scientific name	Family	Common name	Part used	Use
*Apium graveolens*	Apiaceae	Celery		Kidney tonic
*Bauhinia cumanensis */*excisa*	Fabaceae	Monkey step	Bark	Gall stones
*Begonia humilis*	Begoniaceae	Lozeille		Cooling
*Bontia daphnoides*	Myoporaceae	Olive bush	Leaves	Cooling
*Capraria biflora*	Scrophulariaceae	Du thé pays	Leaves	Gall stones, cooling
*Cassia alata*	Fabaceae	Senna	Leaves	Cooling with cloves & ginger
*Chamaesyce hirta*	Euphorbiaceae	Mal nommée		Kidney problems
*Cissus verticillata*	Vitaceae	Blister bush	Vine	Cooling
*Cocos nucifera*	Arecaceae	Coconut	Root	Bladder stones
*Coleus aromaticus*	Lamiaceae	Spanish thyme	Leaves	Cooling
*Commelina elegans*	Commelinaceae	Water grass	Plant	Cooling
*Costus scaber*	Zingiberaceae	Wild cane		Cleans bladder
*Cuscuta americana*	Convolvulaceae	Love vine	Vine	Cooling
*Cynodon dactylon*	Poaceae	Dube		Stoppage of water
*Cyperus rotundus*	Cyperaceae	Nut grass		Cooling
*Desmodium canum*	Fabaceae	Sweet heart bush	Plant	Cooling
*Eleusine indica*	Poaceae	Dead man's grass	Root, Leaves	Urinary
*Entada polystachya*	Fabaceae	Mayoc chapelle		Cooling
*Flemingia strobilifera*	Fabaceae	Kidney bush		Kidney problems
*Gomphrena globosa*	Amaranthaceae	Bachelor button	Leaves	Urinary problems
*Hibiscus sabdariffa*	Malvaceae	Sorrel	Flower & seed	Cleans liver and blood
*Justicia pectoralis*	Acanthaceae	Carpenter grass	Leaves	Cooling
*Kalachoe pinnata*	Crassulaceae	Wonder of the world	Leaves	Cooling, Bladder stones
*Lepianthes peltata*	Piperaceae	Lani bois	Leaves	Tea
*Mimosa pudica*	Fabaceae	Ti marie, mese marie		Cooling, Kidney problems
*Momordica charantia*	Cucurbitaceae	Caraaili	Vine	Cooling
*Musa *species	Musaceae	Banana	Dry leaf	Boil for cooling
*Nopalea cochinellifera*	Cactaceae	Rachette	Joint	Kidney stones
*Peperomia rotundifolia*	Piperaceae	Giron fleur, mowon		Kidney problems
*Peperomia pellucida*	Piperaceae	Shining bush		Cooling
*Petiveria alliacea*	Phytolaccaceae	Mapourite, kudjuruk		Kidney problems
*Pilea microphylla*	Urticaceae	Du thé bethelmay	Leaves	Bladder cleanser
*Pityrogramma calomelanos*	Pteridaceae	Fern		Urinary problems
*Portulaca oleraceae*	Portulacaceae	Pussley	Plant	Cholesterol, shortness of breath
*Ruellia tuberosa*	Acanthaceae	Minny root	Root	Cooling
*Sansevieria guineensis*	Agavaceae	Langue bouef, lash	Leaves	Cooling
*Scoparia dulcis*	Scrophulariaceae	Sweet broom	Plant	Cooling for babies
*Solanum americanum*	Solanaceae	Agouma, gouma	Plant	Cooling, provides iron
*Solanum melongena*	Solanaceae	Melongene	Fruit	Cholesterol
*Stachytarpheta jamaicensis*	Verbenaceae	Vervine	Leaves	Cooling
*Theobroma cacao*	Sterculiaceae	Cocoa	Core	Eat for urinary problems
*Tournefortia hirsutissima*	Boraginaceae	Chigger bush	Leaves	Cooling
*Vetiveria zizanioides*	Poaceae	Vetivert		Urinary problems
*Zea mays*	Poaceae	Corn silk	Stigma	Diuretic

Plants used for high blood pressure, diabetes and jaundice

Twenty-nine plants are used for diabetes and hypertension including four for jaundice.

Multiple-plant remedies are used for several conditions including one used for jaundice which combined white bachelor button (*Gomphrena globosa*), olive bush (*Bontia daphnoides*), small white vere michelle (unidentified), and fine-stemmed rather than thick-stemmed love vine (*Cuscuta americana*).

The following plants are used to treat diabetes: *Antigonon leptopus*, *Bidens alba*, *Bidens pilosa*, *Bontia daphnoides*, *Carica papaya*, *Gomphrena globosa*, *Bixa orellana*, *Catharanthus roseus*, *Cocos nucifera*, *Laportea aestuans*, *Momordica charantia*, *Morus alba*, *Phyllanthus urinaria *and *Spiranthes acaulis*.

*Apium graveolens *is used as a heart tonic and the following plants are used for hypertension: *Aloe vera, Annona muricata*, *Artocarpus altilis*, *Bixa orellana*, *Bidens alba*, *Bidens pilosa*, *Bontia daphnoides*, *Carica papaya*, *Cecropia peltata*, *Citrus paradisi*, *Cola nitida*, *Crescentia cujete*, *Gomphrena globosa, Hibiscus sabdariffa*, *Kalanchoe pinnata*, *Nopalea cochinellifera*, *Morus alba*, *Ocimum campechianum*, *Passiflora quadrangularis*, *Persea americana *and *Tamarindus indicus*. Low blood pressure is treated with *Apium graveolens*.

Jaundice is treated with the following plants (many of which are also listed above): *Bixa orellana*, *Bontia daphnoides*, *Gomphrena globosa *and *Cuscuta americana*.

The ethnomedicinal plants used in Trinidad and Tobago for diabetes are summarised in Table 2.

**Table 2:** Ethnomedicinal plants used for high blood pressure, jaundice and diabetes in Trinidad and Tobago

Scientific name	Family	Common name	Plant part used	Use
*Aloe vera*	Liliaceae	Aloes	Leaf gel	Hypertension
*Annona muricata*	Annonaceae	Soursop	Leaves	Hypertension
*Antigonon leptopus*	Polygonaceae	Coralita	Vine	Diabetes
*Apium graveolens*	Apiaceae	Celery		Heart tonic, Low blood pressure
*Artocarpus altilis*	Moraceae	Breadfruit	Leaves	Hypertension
*Bidens alba/Bidens pilosa*	Asteraceae	Needle grass	Leafy branch	Hypertension, Diabetes
*Bixa orellana*	Bixaceae	Roucou	Leaves, root	Hypertension, Diabetes, Jaundice
*Bontia daphnoides*	Myoporaceae	Olive bush	Leaves	Diabetes, Jaundice, Hypertension
*Carica papaya*	Caricaceae	Papaya	Green fruit	Hypertension, Diabetes
*Catharanthus roseus*	Apocynaceae	White Periwinkle		Diabetes
*Cecropia peltata*	Cecropiaceae	Bois canôt	Leaves	Hypertension
*Citrus paradisi*	Rutaceae	Grapefruit	Peel	Hypertension
*Cocos nucifera*	Arecaceae	Coconut	Shell, flower	Diabetes
*Cola nitida*	Sterculiaceae	Obie seed	Seed	Hypertension
*Crescentia cujete*	Bignoniaceae	Calabash	Leaves	Hypertension
*Cuscuta americana*	Convolvulaceae	Love vine	Vine	Jaundice
*Gomphrena globosa*	Amaranthaceae	Bachelor button	Leaves	Jaundice, Diabetes, Hypertension
*Hibiscus sabdariffa*	Malvaceae	Sorrel	Leaf	Hypertension
*Kalanchoe pinnata*	Crassulaceae	W/world	Leaf	Hypertension
*Laportea aestuans*	Urticaceae	Red stinging nettle	Leaves	Diabetes
*Momordica charantia*	Cucurbitaceae	Caraaili		Diabetes
*Morus alba*	Moraceae	Pawi bush		Diabetes, Hypertension
*Nopalea cochinellifera*	Cactaceae	Rachette	Joint	Hypertension
*Ocimum campechianum*	Lamiaceae	Ti bom	Leaves	Hypertension
*Passiflora quadrangularis*	Passifloraceae	Barbadine	Leaves	Hypertension
*Persea americana*	Lauraceae	Avocado	Leaf	Hypertension
*Phyllanthus urinaria*	Euphorbiaceae	Red seed under leaf		Diabetes
*Spiranthes acaulis*	Orchidaceae	Lapsogen		Early-stage diabetes
*Tamarindus indicus*	Fabaceae	Tamarind	Seed	Hypertension

## Discussion

Non-experimental validation

For each species or genus the ethnomedicinal uses in other countries was examined but will not be presented in the table due to space constraints. Only the most relevant clinical trials are presented. The non-experimental validation is summarised in Tables 3 and 4. Table 4 is an additional file (see Additional file 1).

**Table 3:** Validation of plants used for "cooling", high cholesterol and urinary problems in Trinidad and Tobago

Scientific name	Common name	Use	Validation score
*Apium graveolens*	Celery	Kidney tonic	3 for pain
*Bauhinia cumanensis */*excisa*	Monkey step	Gall stones	3 for pain
*Begonia humilis*	Lozeille	Cooling	2
*Bontia daphnoides*	Olive bush	Cooling	2
*Capraria biflora*	Du thé pays	Gall stones, cooling	3 for pain
*Cassia alata*	Senna	Cooling with cloves and ginger	2
*Chamaesyce hirta*	Malomay, Mal nommée	Kidney problems	3 diuretic; 3 sedative
*Cissus verticillata*	Blister bush	Cooling	3 diarrhoea; 3 gastric ulcers
*Cocos nucifera*	Coconut	Bladder stones	2
*Coleus aromaticus*	Spanish thyme	Cooling	2
*Commelina elegans*	Water grass	Cooling	2
*Costus scaber*	Wild cane	Cleans bladder	data needed
*Cuscuta americana*	Love vine	Cooling	2
*Cynodon dactylon*	Dube	Stoppage of water	2
*Cyperus rotundus*	Nut grass	Cooling	3
*Desmodium canum*	Sweet heart bush	Cooling	3
*Eleusine indica*	Dead man's grass	Urinary	2
*Entada polystachya*	Mayoc chapelle	Cooling	2 data needed
*Flemingia strobilifera*	Kidney bush	Kidney problems	2 data needed
*Gomphrena globosa*	Bachelor button	Urinary problems	2 data needed
*Hibiscus sabdariffa*	Sorrel	Cleans liver and blood	3 hypertension; 3 liver problems
*Justicia pectoralis*	Carpenter grass	Cooling	Data needed
*Kalachoe pinnata*	Wonder of the world	Cooling, Bladder stones	3 jaundice
*Lepianthes peltata*	Lani bois	Tea	Data needed
*Mimosa pudica*	Ti marie, mese marie	Cooling, Kidney problems	2
*Momordica charantia*	Caraaili	Cooling	Data needed
*Musa *species	Banana	Boil for cooling	2
*Nopalea cochinellifera*	Rachette	Kidney stones	2 data needed
*Peperomia rotundifolia*	Giron fleur, mowon	Kidney problems	3
*Peperomia pellucida*	Shining bush	Cooling	3
*Petiveria alliacea*	Mapourite, kudjuruk	Kidney problems	Data needed
*Pilea microphylla*	Du thé bethelmay	Bladder cleanser	2
*Pityrogramma calomelanos*	Fern	Urinary problems	Data needed
*Portulaca oleraceae*	Pussley	Cholesterol, short breath	3 pain; 3 gastroprotective
*Ruellia tuberosa*	Minny root	Cooling	Data needed
*Sansevieria guineensis*	Langue bouef, lash	Cooling	Data needed
*Scoparia dulcis*	Sweet broom	Cooling for babies	3 pain; 3 diuretic
*Solanum americanum*	Agouma, gouma	Cooling, provides iron	2 data needed
*Solanum melongena*	Melongene	Cholesterol	Not proven
*Stachytarpheta jamaicensis*	Vervine	Cooling	Data needed
*Theobroma cacao*	Cocoa	Eat for urinary problems	Data needed
*Vetiveria zizanioides*	Vetivert	Urinary problems	Data needed
*Zea mays*	Corn silk	Diuretic	3 diuretic

## Conclusion

From the 1930s the impact of western medicine in the Caribbean has been that of a dominant paradigm that was not totally accepted, but which offered elements that were selectively appropriated in a process of indigenisation [117,118]. The concepts of 'structural superiority' and 'functional strength' imply that western medicine acquired élite status because of its ability to control diseases (or suppress symptoms), while the folk medicinal system retained functional strength because it was more accessible and available to those isolated communities that existed well into the twentieth century [1,117,118]. However this explanatory model, western medicine, has become the dominant medical system; the main means of establishing whether a technology works and how. The non-experimental validation of the ethnoveterinary medicines was undertaken in recognition of that fact. Validation of traditional medicines is important since most scientists will not use local medicines without some form of validation and they have discouraged the use of these medicines leading to their disuse and eventual loss [1]. This loss is exacerbated by the death of knowledgeable elders. A framework of indigenous knowledge that can interface with science and technology is more likely to influence scientific research agendas and development work, leading to the integration of indigenous knowledge into modern life [1,24].

More data is necessary to evaluate the safety of the following plants used to treat urinary problems, "cooling" and high cholesterol levels: *Costus scaber*, *Cynodon dactylon*, *Entada polystachya*, *Flemingia strobilifera*, *Gomprena globosa*, *Justicia pectoralis*, *Lepianthes pelata*, *Momordica charantia*, *Nopalea cochenillifera*, *Petiveria alliacea*, *Pityrogramma calomelanos*, *Ruellia tuberosa*, *Sansevieriea guineensis*, *Stachytarpheta jamaicensis*, *Theobroma cacao *and *Vetiveria zizanioides*.

Little data was found to support the use of the following plants to treat urinary problems: *Justicia pectoralis*, *Lepianthes pelata*, *Momordica charantia*, *Petiveria alliacea*, *Pityrogramma calomelanos*, *Ruellia tuberosa*, *Sansevieriea guineensis*, *Stachytarpheta jamaicensis*, *Theobroma cacao *and *Vetiveria zizanioides*.

The following plants have established analgesic or sedative effects: *Apium graveolens*, *Bauhinia cumanensis*, *Capraria biflora*, *Chamaesyce hirta *and *Portulaca oleraceae*.

*Chamaesyce hirta*, *Cissus verticillata*, *Kalanchoe pinnata*, *Peperomia *spp.,

*Chamaesyce hirta*, *Cissus verticillata*, *Kalanchoe pinnata*, *Peperomia *spp., *Portulaca oleraceae*, *Scoparia dulcis*, and *Zea mays *have sufficient evidence to support their traditional use for urinary problems, "cooling" and high cholesterol. *Cuscuta americana *also merits more study.

The plants used for hypertension, jaundice and diabetes that may be safe and justify more formal evaluation are *Annona squamosa*, *Aloe vera*, *Apium graveolens*, *Bidens alba*, *Carica papaya*, *Catharanthus roseus*, *Cecropia peltata*, *Citrus paradisi*, *Hibsicus sabdariffa*, *Momordica charantia*, *Morus alba*, *Persea americana*, *Phyllanthus urinaria*, *Tamarindus indicus *and *Tournefortia hirsutissima*. Several of the plants are used for more than one condition and further trials should take this into account. The use of eggplant extract as a hypocholesterolemic agent has some support but needs more study.

It is likely that medical practitioners in Trinidad and Tobago are counter-prescribing the majority of these remedies and should learn more about them.

## Competing interests

The author(s) declare that they have no competing interests.

## Supplementary Material

Additional File 1 Table 4. Non-experimental validation of plants used in Trinidad and Tobago for diabetes and urinary problems. The data provided represents the chemical/pharmaceutical/pharmacological literature of the plant's known chemical constituents to determine the known physiological effects of either the crude plant, related species, or isolated chemical compounds that the plant is known to contain, and also any relevant clinical trials. Click here for file
