# Supplementary material for: Ethnomedicines used in Trinidad and Tobago for urinary problems and diabetes mellitus
Source: J Ethnobiol Ethnomed. 2006 Oct 13;2:45. doi: 10.1186/1746-4269-2-45 (PMC1624823; doi:10.1186/1746-4269-2-45)
Supplement: Additional File 1 — Table 4. Non-experimental validation of plants used in Trinidad and Tobago for diabetes and urinary problems. The data provided represents the chemical/pharmaceutical/pharmacological literature of the plant's known chemical constituents to determine the known physiological effects of either the crude plant, related species, or isolated chemical compounds that the plant is known to contain, and also any relevant clinical trials. [file 1746-4269-2-45-S1.pdf]

Table 4. Non-experimental validation of plants used in Trinidad and Tobago for diabetes and urinary problems

Scientific name	*Clinical study*	#
Aloe barbadensis	*Aloe vera* improved wound healing in STZ diabetic mice. Extracts of aloe gum increased glucose tolerance in diabetic and normal rats. Constant administration of the exudate of the leaves of *Aloe barbadensis* (500 mg/kg PO) gave a significant hypoglycemic effect in alloxan-diabetic mice. Single and chronic administration of the bitter principle (5 mg/kg IP) gave a significant hypoglycemic effect. The dried sap of the plant (half a tsp daily for 4–14 weeks) showed a significant hypoglycemic effect clinically as well as experimentally.	26
Annona squamosa	Doses of aqueous leaf extracts of *Annona squamosa* were given in drinking water to normal and (streptozotocin–nicotinamide induced diabetic) experimental rats for 12 days (250 mg/kg and 500mg/kg). The control was the standard drug glibenclamide (0.25 mg/kg). The aqueous extract lowered the fasting plasma glucose levels (P<0.05). A significant difference was also seen in serum insulin levels, serum lipid profile, liver glycogen levels and in pancreatic TBARS levels. The antidiabetic activity was not dose dependant. There was no toxicity found in the aqueous extract and the antidiabetic claims of *Annona squamosa* were supported.	27
A. leptopus	*Antigonon leptopus* plant has antithrombin activity.	28
Apium graveolens	*Apium graveolens* (celery) aqueous extract was found to have an antihyperlipidemic property which was not due to 3-n-butylphthalide (BuPh) (BuPh was previously reported to produce the lipid-lowering action in celery). Apigenin, isolated from *Apium graveolens*, relaxes rat thoracic aorta. *Apium graveolens* has antiinflammatory activity and antinociceptive action. The latter may be due to the presence of volatile oils, flavonoids and resins. The use of the plant extract for inflammation, pain and spasmodic colic can be justified.	1, 29
Artocarpus altilis (syn. *Artocarpus communis*)	A geranylated chalcone was isolated from leaves of *Artocarpus incisus* and it showed potent 5-alpha-reductase inhibitory activity. An extract of the leaves of *Artocarpus altilis* exerted a negative inotrophic effect on rat myocardium.	1
Bauhinia species	A new flavonol glycoside extracted from the roots of *Bauhinia variegata* showed anti-inflammatory activity. The crude methanolic extract of the leaves of Brazilian *Bauhinia microstachya* showed analgesic properties.	1, 30, 31
Begonia species	Four *Begonia* species have antimicrobial activity. One of these four (*Begonia heracleifolia*) and two other species have anti-tumour activity. The aqueous extract of *Begonia malabarica* showed activity against nine human pathogenic bacteria but not against *Vibrio parahaemolyticus*.	1, 32

Bidens alba	*Bidens alba* L. var. *radiata* Schultz-Bip, (synonym) *Bidens pilosa*, contains polyacetylenes, which inhibit pathogenic microorganisms and flavonoids and are active anti-inflammatory agents. *Bidens pilosa* aqueous leaf extract possesses aortic smooth muscle relaxant activity. A butanol fraction of *Bidens pilosa* prevented diabetes in non-obese diabetic (NOD) mice. The antidiabetic action is possibly due to two polyacetylenic glucosides.	1, 33
Bixa orellana	*Bixa orellana* contains annatto which was responsible for the hypoglycaemic episodes seen in the oral glucose tolerance test (OGTT) that was performed on dogs. This was mediated by an increase in plasma insulin concentration. Bixin has a weak effect on rat liver monooxygenases.	1, 34 35
Bontia daphnoides	The use of *Bontia daphnoides* for jaundice in Trinidad may be related to its use for urinary problems. *Bontia daphnoides* may have an Australian ancestral lineage. *Bontia daphnoides* contains (-)-epingaione, a sesquiterpene furan. A herbarium note was found that reported *Bontia daphnoides* fruit can be used as an antidote to the very poisonous *Hippomane mancinella*.	1, 16, 37, 38
Capraria biflora	The aqueous extract (50–200 mg kg^{-1}) of dried leaves of *Capraria biflora* had analgesic properties.	1, 39, 40
Carica papaya	The fruit juice of unripened fruit of *Carica papaya* may contain antihypertensive agent(s) which exhibit(s) alpha-adrenoceptor activity.	41, 42
Cassia alata	The methanol extracts of leaves, flowers, stem and root barks of *Cassia alata* showed broad spectrum antibacterial activity. *Cassia alata* leaf extract had no effect on glucose levels in normoglycemic rats, but it reduced the blood sugar levels in streptozotocin-induced hyperglycemic rats.	1, 43
Catharanthus roseus	The leaf juice of *Catharanthus roseus* produced a dose-dependent hypoglycemia in normal rabbits. A dose-dependent reduction in blood glucose was also observed in alloxan-induced diabetic rabbits treated with *Catharanthus roseus*. Active principles are three alkaloids: leurosine, vindoline and vindolinine which are more potent than tolbutamide as hypoglycaemic agents.	1,44
Cecropia species	The stems and leaves of *Cecropia* cf.*obtusifolia* Bertol., inhibited the [3H]-AT II binding (angiotensin II AT1 receptor) more than 50%. The ethanolic extracts of the leaves of *Cecropia* cf. *obtusifolia* Bertol., and the stem of *Cecropia* cf. *obtusifolia* Bertol showed high inhibition of the [3H] BQ-123 binding (endothelin-1 ET(A) receptor) in a preliminary screening. The aqueous extracts from the leaves of *Cecropia obtusifolia* have low toxicity, a substantial central depressor effect and analgesic activity, significant motor incoordination and muscle relaxant activity and a peripheral analgesic effect. The extracts also showed a topical and systemic anti-inflammatory effect. These results could justify the popular use of *Cecropia obtusifolia* in rheumatic and kidney inflammation	1, 45, 46

	pathologies. *Cecropia glazioui* Sneth has antihypertensive-hypotensive action which may be related to blockade of voltage-gated calcium channels in vascular smooth muscle, while the positive inotropic/chronotropic and broncho-relaxant effects may be produced by a beta-adrenergic activity.	
Chamaesyce hirta syn. *Euphorbia hirta*	The active component(s) in the aqueous extract of *Chamaesyce hirta* leaf had a diuretic spectrum comparable to that of acetazolamide, a standard diuretic drug, increasing both electrolyte excretion and urine output. Ethanolic extracts of the leaves also induced diuresis in rats. This study justified the traditional use of *Chamaesyce hirta* by the Swahilis and Sukumas as a diuretic agent. The lyophilised aqueous extract of *Chamaesyce hirta* has sedative activity at high doses (100 mg of dried plant/kg) without toxic effects. This study validated the traditional use of *Chamaesyce hirta* as a sedative.	1, 47, 48
Cissus sicyoides	The aqueous extract from *C. sicyoides* had an anti-inflammatory effect. *Cissus rubiginosa* is used as an antidiarrhoeal agent in Congolese folk medicine. The methanolic and aqueous extracts derived from *Cissus rubiginosa* have antibacterial activity possibly due to tannins, phenolic compounds and flavonoids and the ethnomedical use of *Cissus rubiginosa* as an anti-diarrhea agent is defensible. *Cissus quadrangularis* (methanolic extract) is comparable to sucralfate in the treatment of gastric ulcers and should be tested for the treatment of peptic ulcer disease.	1, 49, 50, 51
Citrus species	The contents of the bioactive compounds in the pummelo-grapefruit hybrid juice named Israeli Jaffa Sweetie and their effect on people suffering from hypercholesterolemia were studied in a randomized controlled trial with 72 hypercholesterolemic patients, aged 43-71 years, after coronary bypass surgery. Diet supplemented with this juice positively influenced serum lipid, albumin, and fibrinogen levels and their antioxidant capacities; the juice thus has the potential to help hypercholesterolemic patients.	1, 52
Cocos nucifera	*Cocos nucifera* kernel is reported to contain a mannan. The fiber husk of coconut is rich in catechins (polyphenols with antioxidant activity); it has anti-bacterial and anti-viral activity and was tested for anti-proliferative activity and has a role to play in controlling hypertension.	1, 53, 54, 55
Cola nitida	*Cola nitida* nuts contain a heart stimulant called kolanin, and also contain caffeine, theobromine and quinine which are associated with increased blood pressure.	1
C. aromaticus	Leaves of *Coleus aromaticus* have antioxidant activity.	56
Commelina elegans	An aqueous extract of *Commelina communis* alleviated hyperglycemia caused by maltose or starch loading in normal and Streptozotocin (STZ)-induced diabetic mice with better efficacy than that of acarbose. In addition, prolonged administration of *Commelina communis* normalized hyperglycemia in STZ-induced diabetic mice. Therefore *Commelina communis* has potential for use in	1, 57

	the management of non-insulin-dependent diabetes.	
Crescentia cujete	*Crescentia cujete* contains alkaloids, tannins and flavonoids as its antimicrobial constituents. *Crescentia alata* methanolic (MeOH) extract was evaluated *in vivo* and had a significant anti-inflammatory activity at the highest dose tested possibly due to kaempferol.	1
Cuscuta americana	At high doses *Cuscuta* species can cause fatal gastro-intestinal toxicity. *Cuscuta reflexa* is used in Uttar Pradesh India to treat jaundice and in Uttaranchal to treat 31 ailments. The methanol extract of *Cuscuta reflexa* Roxb. stem (MECR) was evaluated in multiple weekly doses of MECR (25, 50, 75 mg/kg, i.p.) on liver and kidney functions in mice. MECR at medium and high dose levels increased serum transaminase (SGOT and SGPT), non-protein nitrogen (NPN) and plasma cholesterol significantly suggesting a negative effect on the liver. Serum alkaline phosphatase and total bilirubin in the MECR treated mice were also increased by the moderate dose. The high dose did increase creatinine levels significantly. Low doses of MECR (25 mg/kg, i.p.) did not produce any noticable change in liver and kidney functions. The methanolic extract (ME) of *Cuscuta reflexa* stem suppressed ovarian steroidogenesis in mice.	1, 59-62
Cuscuta reflexa	The petroleum ether extract of *Cuscuta reflexa* Roxb. stem has significant analgesic properties. Steroids are the active compounds.	61, 62
Cynodon dactylon	*Cynodon dactylon* is one of the rejuvenative plants with antioxidant activity used in the Ayurvedic system to prevent and treat degenerative diseases (*Rasayana*). *Cynodon dactylon* decoction caused a significant decrease in hyperglycemia in healthy rabbits with induced temporary hyperglycemia.	63
Cyperus rotundus	*Cyperus scariosus* root comprised one part of a polyherbal ayurvedic preparation that provided partial protection to rats with cisplatin-induced renal toxicity. It has shown cytoprotective effect against ethanol induced ulceration in rats. A polyherbal formulation, containing *Aegle marmeloes*, *Coriandrum sativum*, *Cyperus rotundus* and *Vetiveria zinzanioides* was effective in indomethacin-induced enterocolitis in rats as well as acetic acid-induced colitis in mice (models of inflammatory bowel disease). *Cyperus rotundus* and its sesquiterpenes may be a significant contributor to the activity of the formulation.	65, 66
Desmodium adscendens	*Desmodium styracifolium* was used as one component in a twelve-herb mixture used to successfully treat bovine urolithiasis. The ethanol extract of *Desmodium canum* roots contains antimicrobially active prenylated isoflavanones (desmodianones). *Desmodium adscendens* butanolic extract inhibits contraction of the ileum and trachea in guinea pigs. An aqueous extract of *Ocimum canum* is used by Ghanaians to manage diabetes mellitus. *In vivo* modulation of levels of fasting blood glucose by *0. canum* extract was evaluated in type-II diabetes mellitus	1, 67-69

	using the C57BL/KsJ db/db genetically diabetic animal model, and its effects on glucose-stimulated insulin release in vitro were monitored using isolated rat pancreatic beta-islet cells. The results showed that fasting blood glucose levels and body weight decreased significantly ($p < 0.05$) in diabetic and non-diabetic C57BL/KsJ mice, which were administered aqueous extract of *0. canum*. Addition to the medium of *Desmodium adscendens*, a plant preparation used to manage inflammatory disorders inhibited insulin secretion by the pancreatic beta-islet cells. Aqueous extracts of *Desmodium styracifolium* were studied in rats *in vivo* and *in vitro*. *Desmodium styracifolium* produced two successive hypotensive actions: the first one via cholinergic receptor stimulation, while the second one potentiated by blockades of autonomic ganglion and alpha-adrenoceptor.	
Eleusine indica	Consumption of finger millet based diets resulted in significantly lower plasma glucose levels. Factors may be its higher fiber content compared to rice and wheat or the presence of antinutritional factors in whole finger millet flour which are known to reduce starch digestibility and absorption.	72
Entada polystachya	Extracts of *Entada abyssinica* (stem bark), showed activity against various *Candida* species. *Entada africana* exhibited antimicrobial activity against *Vibrio cholerae*. *Entada abyssinica* leaves have antiviral activity.	73-75
Flemingia strobilifera	A new salicylic acid derivative and four new isoflavones were obtained from the stem bark of *Flemingia paniculata*. *In vitro* vermifugal activity in *Flemingia vestita* may be due to the isoflavone genistein.	76, 77
Gomphrena globosa	The ethnomedicinal use of *Gomphrena globosa* for jaundice may be related to its use for urinary problems. The ethanolic extract and pure compounds of *Gomphrena celosioides* have antibacterial activity.	1, 78
Hibiscus sabdariffa	A controlled and randomized clinical trial compared the antihypertensive effectiveness and tolerability of a standardized extract from *Hibiscus sabdariffa* with captopril. *Hibiscus sabdariffa* was able to decrease the systolic blood pressure (BP) from 139.05 to 123.73mm Hg (ANOVA $p < 0.03$) and the diastolic BP from 90.81 to 79.52mm Hg (ANOVA $p < 0.06$). At the end of the study there were no significant differences between the BP recorded in both treatment groups (ANOVA $p > 0.25$). The experimental treatment produced a natriuretic effect.	79
Hibiscus sabdariffa	The average consumption of 150-180 mg/kg per day of *H. sabdariffa* infusion may be safe; higher doses may have a negative effect on the liver. The effect of the water extract of the dried flowers of *Hibiscus sabdariffa* L. and *Hibiscus* anthocyanins (HAs) (natural pigments found in the dried calyx of *H. sabdariffa*) on paracetamol-induced hepatotoxicity in rats was studied. The high oral dose of 200 mg/Kg for 5 consecutive days restored the indicators of liver damage to normal. Lower doses (50 and 100 mg/kg) were ineffective.	79-81

Justicia pectoralis	*Justicia pectoralis* contains coumarins (dihydrocoumarin and umbelliferone), betaine and 3-(2-hydroxyphenyl) propionic acid. Coumarin and umbelliferone are major constituents of the plant and have the ability to relax smooth muscle.	1, 82
Kalanchoe pinnata	The leaf juice concentrate and ethanolic extract of *Kalanchoe pinnata* provide hepatoprotection, but the concentrate is more effective. The plant concentrate is very effective in decreasing the elevated level of serum bilirubin suggesting that it can be used in the acute condition of jaundice. The juice of the leaves and the ethanolic extract of the marc of *Kalanchoe pinnata* Pers. (synonyms: *Bryophyllum calycinum* Salisb. Parad. Lond., *B. pinnatum* Kurz.) (family Crassulaceae) left after expressing the juice were studied in rats against CCl4-induced hepatotoxicity. The juice had a greater hepatoprotective effect than the ethanolic extract.	1, 83
Laportea aestuans	The antidiabetic and hypolipidaemic effects of a methanol/methylene-chloride extract of the aerial parts of *L. ovalifolia* were investigated, in normal rats and rats with diabetes induced by the intraperitoneal injection of alloxan (at 150 mg/kg bodyweight). In the diabetic rats, 2 weeks of daily, intragastric treatment with the *L. ovalifolia* extract produced a significant reduction in the fasting serum glucose concentrations and lowered the serum concentrations of total cholesterol and increased the serum concentration of HDL cholesterol. *L. ovalifolia* may possess antidiabetic and hypolipidaemic properties.	1, 84
Mimosa pudica	*Mimosa pudica* exhibited antimicrobial activity against *Vibrio cholerae*. It did not prevent bladder stone deposition or dissolve preformed stones (experimental urolithiasis in male and female rats).	85
Momordia charantia	*Momordia charantia* can control glucose levels in chemically induced mild to severe models of diabetes mellitus in rodents and it may act by stimulating kinases involved in peripheral utilization of glucose. Diabetic complications are the major cause of morbidity and mortality in diabetes mellitus. Use of *Momordia charantia* has produced positive outcomes in diabetic complications like diabetic nephropathy, fructose induced insulin resistance and cataracts.	1, 86, 87
Momordia charantia	In normal mice intraperitoneal administration of *Momordica charantia* aqueous extract improved glucose tolerance in normal mice after eight hours and reduced the level of hyperglycaemia in streptozotocin diabetic mice by 50% after five hours. *Momordica charantia* fruit juice acts like insulin to exert its hypoglycaemic effect and also stimulates amino acid uptake into skeletal muscle cells. *Momordica charantia* fruit was studied with oral hypoglycemis in Non-Insulin Dependent Diabetes Mellitus (NIDDM) patients. The extract acts in synergism with oral hypoglycemics and potentiates their hypoglycemia in NIDDM.	1, 86, 87

Morus alba	The hypoglycaemic activity of a 20% dried leaf infusion of *Morus alba* was not verified in alloxan and streptozotocin induced hyperglycaemic rats. Chronic subcutaneous administration of the extract of leaves of *Morus alba* to rabbits led to degranulation of beta-cells of the Langerhans islets. Leaves of *Morus alba* inhibit digestion and could be used as an ingredient in health foods and in foods that help to prevent diabetes. The hypoglycemic activity of the flavonoids rich fraction of 70% alcohol extract of the Egyptian *Morus alba* root bark was evaluated after its oral administration to streptozotocin-induced diabetic rats. The extract may protect pancreatic beta cells from degeneration and diminish lipid peroxidation.	88-90
Musa sapientum	The antihyperglycemic effect of ethanolic extract of flowers of *Musa sapientum* (Musaceae) was studied in alloxan induced diabetic rats. Oral administration of the ethanolic extract showed significant ($p < 0.001$) blood glucose lowering effect at 200 mg/kg in alloxan induced diabetic rats (120 mg/kg, i.p.). The methanolic extract of mature, green fruits of *Musa paradisiaca* (MEMP) was examined in normal (normoglycemic) and streptozotocin (STZ)-treated, diabetic (hyperglycemic) mice, using chlorpropamide as the reference antidiabetic agent. MEMP (100-800 mg/kg p.o.) induced significant, dose-related ($p < 0.05$-0.001) reductions in the blood glucose concentrations of both normal and diabetic mice and showed hypoglycemic activity. The folkloric use of the plant in the management of adult-onset, type-2 diabetic mellitus is supported.	91, 92
Nopalea cochinellifera	*Nopalea cochinellifera* is closed related to prickly pear which is used by Pima Indians as treatment against diabetes mellitus and for its lipid lowering properties. Disturbances in glucose and lipid metabolism are associated with impaired platelet function. Platelet function is one of several factors that contribute to the initiation and progression of atherosclerosis. Prickly pear's beneficial activity on the cardiovascular system may come from decreasing platelet activity and hence improving haemostatic balance. *Nopalea cochinellifera* stems tested in oral glucose tolerance test in mice produced an increase in blood glucose levels.	93, 94
Ocimum campechianum	*Ocimum sanctum* fixed oil produced a hypotensive effect in the anaesthetised dog, which may be due to its peripheral vasodilatory action. The oil increased blood-clotting time and percentage increase was comparable to aspirin and this could be due to inhibition of platelet aggregation. Results of a randomized, placebo-controlled, crossover, single blind clinical trial of leaf extract of *Ocimum album* showed a significant decrease in fasting, post-prandial blood levels and mean total cholesterol levels in treated subjects as compared to controls. Constituents of *O. sanctum* leaf extracts have stimulatory effects on physiological pathways of insulin secretion	1,95, 96

	which may explain its antidiabetic activity.	
Passiflora quadrangularis	*Passiflora quadrangularis* whole plant contains nor-epinephrine and 5-hydroxytryptamine; a cyclopropane triterpene glycoside (quadranguloside) was isolated from the leaves. *Passiflora quadrangularis* contains an angiotensin converting enzyme inhibitor and aldose reductase enzyme inhibitor and could be studied for a novel anti-hypertensive agent.	97
Peperomia pellucida	The methanol extract of *Peperomia pellucida* aerial parts (containing anthraquinones, cardiac glycosides and tannins) showed significant analgesic activity. Aqueous extracts of dried aerial parts of *Peperomia pellucida* have anti-inflammatory activity and an analgesic effect at a dose level of 400 mg/kg and low toxicity. *Peperomia pellucida* lowers uric acid in the blood and was endorsed by the Department of Health in the Philippines.	1, 98-103
Persea americana	Intravenous administration of methanol and aqueous extracts of *Persea americana* to anaesthetised normotensive rats produced a fall in mean arterial blood pressure which lasted less than five minutes. The short duration of this effect may indicate rapid metabolism of the active principles (steroid and triterpene glycosides).	1
Phyllanthus urinaria	A methanolic extract of *Phyllanthus amarus* had potential anti-oxidant activity. The extract reduced the blood sugar in alloxan diabetic rats by the 4th hour by 6% at a dose level of 200 mg/kg body wt and 18.7% at a concentration of 1000 mg/kg body wt. Continued administration of the extract for 15 days produced significant ($P < 0.001$) reduction in blood sugar. A one week treatment with the aqueous extract of *Phyllanthus amarus* did not lower FBG or postprandial blood glucose in untreated Non-Insulin Dependent Diabetes Mellitus (NIDDM) patients. In a clinical observation, oral administration of a preparation of the whole plant of *Phyllanthus amarus* (syn. *Phyllanthus niruri*) (5 gm/day in divided doses) for 10 days to 9 mild hypertensives (four subjects with diabetes mellitus) reduced blood glucose (5–50 mg) in diabetic as well as non-diabetic subjects and also reduced systolic blood pressure.	1, 104
P. microphylla	*Pilea microphylla* was active against *Staphylococcus aureus*.	1
Pityrogramma calomelanos	*Pityrogramma calomelanos* methanolic extract has cytotoxic properties.	1
Portulaca oleracea	*Portulaca pilosa* exerts some renal effects that do not include diuresis. *Portulaca oleracea*, *Portulaca grandiflora* and *Portulaca oleracea* L. subsp *sativa* (Haw.) Celak have analgesic and antiinflammatory activities comparable to synthetic drugs. *Portulaca oleracea* aqueous and ethanolic extracts showed a dose-dependent reduction in severity of ulcers induced by HCl or absolute alcohol. The highest dose of extracts exerted similar activity to sucralfate. This gastroprotective activity justifies its use for gastrointestinal disease.	1, 105-107

Ruellia species	*Ruellia patula* yielded two lignan glycosides.	1
Sansevieria guineensis	*Sansevieria guineensis* originates in South Africa. The methanol extract of the whole plant of *Sansevieria trifasciata* yielded 12 steroidal saponins and four pregnane glycosides.	1
Scoparia dulcis	Oral administration of an aqueous extract of *S. dulcis* plant (200 mg/kg of body weight) to streptozotocin diabetic rats for 6 weeks proved that *S. dulcis* had an antihyperlipidemic action in normal and experimental diabetic rats in addition to its antidiabetic effect.	108
Solanum melongena	In two clinical experiments *Solanum melongena* infusion (equivalent to 83g of eggplant fruit) reduced the blood levels of total and LDL cholesterol and of apolipoprotein B in humans. This effect was modest and transitory. No effect of eggplant (containing polyphenols and steroidal saponins) on cholesterol metabolism and atherogenesis in LDLR(-/-) mice was seen after 12 weeks in a more recent study with mice. The eggplant extract (fruit blended in water and given ad lib.) did not act as a hypocholesterolemic agent. *Solanum surrattense* was included in a twelve-herb mixture used to effectively treat bovine urolithiasis.	1, 109-110
Spiranthes acaulis	*Spiranthes autumnalis* and *Spiranthes diuretica* are used as depuratives, tonics and diuretics.	1
Stachytarpheta jamaicensis	The analgesic effect of *Stachytarpheta jamaicensis* was evaluated in rats and showed a lesser effect than morphine.	1
Tamarindus indica	*Tamarindus indica* aqueous extract showed protective activity against lipid peroxidation. The antidiabetogenic effect of aqueous extract of seed of *Tamarindus indica* on streptozotocin-induced diabetic rat in duration dependent fashion was established. *Tamarindus indica* may have beneficial effects in type-I diabetes mellitus.	111
Theobroma cacao	Cocoa extract was tested on serum glucose levels and lipid profiles in streptozotocin-diabetic rats and showed dose-dependent hypoglycaemic and hypocholestrolemic effects on serum glucose levels and lipid profiles, respectively. Cocoa inhibits diabetes-induced cataract formation in rats with diabetes induced by streptozotocin (STZ).The active compound may be the proanthocyanidins which have antioxidative activity.	112, 113
Tournefortia hirsutissima	*Tournefortia hirsutissima* processed in the traditional way showed an antihyperglycaemic effect in temporarily hyperglycemic rabbits validating its ethnomedicinal use in diabetes mellitus control.	114
Vetiveria zizanioides	*Vetiveria zizanioides* has antibacterial properties; and potential in combating intestinal or other pathogens. *Vetiveria zinzanioids* has carminative and stomachic actions that might be useful in gastrointestinal disturbances.	115
Zea mays	Studies on *Zea mays* have shown that it has *in vivo* diuretic and hypotensive activity. Corn silk contains amines, fixed oils, saponins, tannins, bitter glycosides, allantoin, cryptoxanthin, flavone and phytosterols including beta-	1, 116

	sitosterol and stigmasterol. The last two compounds are known to have antiinflammatory activity *in vivo* and may have a beneficial effect in treating prostate problems.	
